# Tongue Muscle Training App for Middle-Aged and Older Adults Incorporating Flow-Based Gameplay: Design and Feasibility Pilot Study

**DOI:** 10.2196/53045

**Published:** 2025-01-09

**Authors:** Kuan-Chu Su, Ko-Chiu Wu, Kuei-Ru Chou, Chia-Hsu Huang

**Affiliations:** 1 College of Design, National Taipei University of Technology, Taipei, Taiwan; 2 Department of Interaction Design, National Taipei University of Technology, Rm.701-4, Design Building, No.1, Sec.3, Chung-hsiao E. Rd, Taipei, 10608, Taiwan, 886 912-595408, 886 2-87732913; 3 School of Nursing, Taipei Medical University, Taipei, Taiwan

**Keywords:** exergame, mobile app, flow, self-care, feasibility, older adults, dysphagia, tongue exercises

## Abstract

**Background:**

Complications due to dysphagia are increasingly prevalent among older adults; however, the tediousness and complexity of conventional tongue rehabilitation treatments affect their willingness to rehabilitate. It is unclear whether integrating gameplay into a tongue training app is a feasible approach to rehabilitation.

**Objective:**

Tongue training has been proven helpful for dysphagia treatment. Following the development of a tongue training app, a feasibility trial aimed to identify physiological and psychological factors that affect user and flow experience and explored whether training specialized muscles could produce a flow experience for optimal immersion. We aimed to provide a useful tool for medical rehabilitation so that older adults could retain tongue muscle flexibility.

**Methods:**

After consulting professional nurses, we developed a mobile gaming app for middle-aged and older adults to train their tongue muscles. This pilot study used an image recognition system to detect the tongue movements of 32 healthy middle-aged and older adults (7 males, 21.9%; 25 females, 78.1%) during 3 game training tasks, each requiring different reaction speeds. Their physiological and psychological signals, as well as the results of the Flow State Scale 2 (FSS2) questionnaire, were used for correlation analysis regarding relevant flow experiences to establish and evaluate the feasibility of our method.

**Results:**

Through exploratory factor analyses, a 2-factor (operation and immersion) structure was confirmed to have an adequate model fit (*χ²*_36_=448.478; *P*<.001; Kaiser-Meyer-Olkin=0.757) and internal consistency reliability (Cronbach α=0.802). The slow, medium, and fast levels all significantly affected the FSS2 score for operation (*P=*.001), the National Aeronautics and Space Administration Task Load Index (*P*<.001), and flow distance (*P*<.001). K-means clustering revealed that participants could be further categorized into 3 groups. Through the analysis of changes in the participants’ physiological and psychological signals for each given task, Pearson correlation indicated that changes were primarily related to flow distance. For the 12 indicators measured in this study, the low, medium, and high operation groups showed significance in 58% (7/12), 50% (6/12), and 25% (3/12) of the indicators, respectively. Similarly, the low, medium, and high immersion groups had changes in 50% (6/12), 33% (4/12), and 17% (2/12) of indicators, respectively.

**Conclusions:**

Our research supports the further development of a gaming app to aid older adults with tongue muscle training and measure flow using physiological and psychological signals to enhance training accuracy and feasibility. Next, we aim to conduct a randomized pilot trial, improve app functions, offer alternative rehabilitation options, and encourage long-term participation. Future goals include enhancing long-term efficacy, diversifying training modes, and adding a multiuser interactive option for an added challenge.

## Introduction

### Background

Compared with desktop-based platforms, mobile phones can provide on-demand leisure and game time [1], in addition to being a handy tool for daily life [2]. Mobile health has become a promising tool to assist older adults with self-care through health and personal care education, medication compliance support, dietary restriction support, setting exercise goals, stress reduction strategies, calling for assistance, and gaining skills for self-management [3]. Mobile health further provides patients with the ability to share data with caregivers and establishes a simple but proactive framework for health management [4].

Dysphagia can lead to rapid aging, disease, and weakened oral expression skills. In older adults, dysphagia is accompanied with a risk of aspiration pneumonia [5-8]. These effects and symptoms affect social activities as well as reduce the dignity and self-esteem of patients [9]. Therefore, the prevention of dysphagia and reduction of potential symptoms are critical.

### Disease Prevention Through Tongue Exercise

Studies have used oral diadochokinesis assessments together with the 10-item Eating Assessment Tool (EAT-10) to evaluate swallowing function [10]. To reduce swallowing impairments in older individuals, the use of speech, tongue-resistance exercises, and head-raising exercises have been noted [11-13]. Tongue-strengthening training devices or exercises [14] are intended to improve swallowing [15-17]. In general, there are 3 different tongue-training methods: stimulus-response therapeutic tongue exercises, playing computer games with the tongue using a tongue drive system, and tongue-protrusion tasks. Findings derived from these tongue-training methods suggest a differential effect of tongue-training paradigms on training-induced cortical plasticity and subject-based scores of fun, motivation, and pain in healthy participants [18]. In addition, exergame training can result in neuroplasticity and cognitive improvement for older adults who are institutionalized [19], while increasing cognitive and physical function in healthy individuals. Exergame training incorporating both cognitive engagement and physical activity exerts greater benefits than cognitively engaging video game training alone [20].

Incorporating gameplay into rehabilitation and training can make such regimens more interesting. Most exergames have been used to improve balance, reduce the effects of Parkinson disease [21], alleviate depressive symptoms in adults [22], and also improve overall fitness [23]. To date, however, there have been very few studies regarding tongue muscle training.

### Roles of Physiological Signals

Having a higher heart rate variability (HRV) is a biomarker reflecting autonomic function and is associated with a greater emotional well-being [24]. It is known that the effect of transcutaneous auricular vagus nerve stimulation on HRV is not regulated by the duration of stimulation. In fact, changes in HRV occur most substantially at the beginning of stimulation [25]. Physiological signals such as pulse, respiration, blood pressure, heart rate, and body temperature can be collected by physiological sensors [26,27]. For example, Garmin device monitors can collect accurate data on heart rate and the number of steps taken during the day [28]. There are also biosensors, which can recognize audio features [29], facial expressions [30], body gestures [31], and even touches on sensitive screens [32]. Furthermore, biosensors can be useful for the detection of emotions by monitoring autonomic nervous system activity [33,34]. Heart rate is a common measurement for cardiovascular strain during training and can provide a visualization of a participants’s training data as well as inform detailed recommendations for training. Heart rate recordings can be performed precisely and in a noninvasive way, which reduces the need for specialized equipment. Exercising according to defined heart rate zones is already well established in professional and recreational endurance training [35]. To a large extent, HRV is modulated by stimulating sympathetic and repressing parasympathetic influences of the autonomic nervous system [36]. It is also known that age and sex have varying effects on heart rate [37].

Several important indicators of variability include heart rate, the root mean square of successive differences (RMSSD), the natural logarithm of the RMSSD (lnRMSSD), the SD of the NN interval (SDNN), low frequency power (LFP), high frequency power, the low frequency to high frequency (LF/HF) ratio, the number of pairs of successive NN intervals that differ by more than 50 milliseconds (NN50), and the proportion of the NN50 divided by the total number of NN intervals (PNN50) [38,39]. However, the complex interactions among physiological and psychological signals mean that they are rarely discussed and studied together in game mechanics.

### Flow Definition and Measurement Questionnaire

Serious games, which are games designed for a purpose other than pure entertainment, can increase patient motivation. Meeting the needs of an individual is often a precursor of a flow state, which is a crucial yet often overlooked feature of serious games [40,41]. Flow is an immersive experience characterized by an optimal balance between one’s current skills and the level of challenge [42-44]. It enables an individual to become highly absorbed, forget the passing of time, and enter a psychological state of ecstatic happiness and contentment. Flow encompasses the following eight dimensions: (1) a combination of challenge and skill, (2) the merging of actions with awareness, (3) clear task goals, (4) direct and immediate feedback, (5) concentration on the task at hand, (6) a sense of control, (7) a loss of self-consciousness, and (8) a perception of the transformation of time [45]. Subsequent studies have added the dimensions of learning and positive subjective experiences [46]. Skills and the degree of learning increase with experience, whereas attention, challenge, the sense of presence, flow, and exploratory behavior decrease with experience [47]. We found that generating feedback, challenge, and reward mechanisms in games can increase user interest. The qualities of the user experience generated in a game, such as hedonic quality and pragmatic quality, which can be determined using the net promoter score (NPS) and the User Experience Questionnaire (UEQ), can lead to the exploration of potential market opportunities [48,49].

The National Aeronautics and Space Administration Task Load Index (NASA-TLX) questionnaire is used to assess perceived workloads and determine the effectiveness of gaming tasks [50]. For the measurement of flow experience, the effectiveness of the Flow State Scale 2 (FSS2), the Dispositional Flow Scale 2, flow distance (FD), EGameFlow, and the Game Experience Questionnaire have been confirmed [24,51]. Among them, FD can be divided into 3 levels to distinguish the current state of experience: anxiety, flow, and boredom [52].

For flow during exercise, an electroencephalogram is often used to investigate the influence of music tempo (fast, slow, or no music control) on flow. An analysis of data collected using the short FSS2 revealed that music tempo exerts a significant impact on subjective experiences and objective physiological characteristics. Higher subjective flow levels have been observed in participants listening to fast-tempo music while walking briskly, showing that fast tempos are conducive to movement flow. These findings demonstrate the benefits of music during sports training to improve training effectiveness [53]. The evidence above highlights that physiological signals can be measured during exercise, and the results of flow questionnaires can reveal whether the user has entered a state of immersion. However, flow states during tongue muscle training have rarely been studied.

### Study Aims

The purpose of this study was to evaluate our mobile health app, which incorporates gameplay for specialized tongue muscle training. The goal of the app is to enable older adults to rehabilitate unassisted and immerse themselves in the process, optimizing their flow. To determine the feasibility of integrating gameplay, we conducted a small, randomized pilot trial by recruiting 32 healthy middle-aged and older users and set up 3 training tasks at different speeds. Since the analysis of patients with dysphagia may be related to more complex variables, this study included healthy individuals for simplicity in the pilot run. This app, by combining tongue exercises with interactive games, could make the process of rehabilitating tongue muscles more interesting and engaging, emphasizing skill training rather than strength training and focusing on retaining users instead of short-term entertainment. The findings derived from our study could serve as a valuable reference for the next stage of development and evaluation. Our aim is to created an app that can help to alleviate and mitigate dysphagia-related conditions and enable older adults to maintain a healthy life.

## Methods

### Overview

The study was conducted in two phases. In the first phase, the study team designed and integrated the game into the tongue app. In the second phase, the study team evaluated the feasibility and acceptability of the integrated game using a single-arm trial.

### Phase 1: Game Design and Development

Registered professional nurses at Taipei Medical University designed the training movements used in the game based on the tongue exercises in the Guidance Manual for Care and Guidance of Eating and Swallowing Difficulties [54], including 6 tongue stretching exercises (Table 1). Once experts had confirmed the accuracy of the tongue exercises, we designed several game icons for the tongue movements, including up, down, left, right, close, and open.

**Table 1.** Descriptions of training movements designed by experts for a mobile game app.

No.	Explanation	Movement segments (image recognition definition)
A1	Extend tongue out of the mouth as far as possible and then retract. Repeat 5 times.	Extend tongue, retract, and close mouth.
A2-1, A2-2	Extend tongue to right corner of mouth as far as possible and then extend to left corner. Repeat 10 times.	Extend tongue to the right, retract, extend tongue to the left, retract, and close mouth.
A3-1, A3-2	Open mouth as much as possible, and circle tongue along lips clockwise. Repeat 10 times.	Open mouth, extend tongue, make a circle, and close mouth.
A4	Push tongue hard against upper front teeth and hold for 10 seconds. Repeat 10 times.	Extend tongue, tongue up, and close mouth.

**Figure 1.**
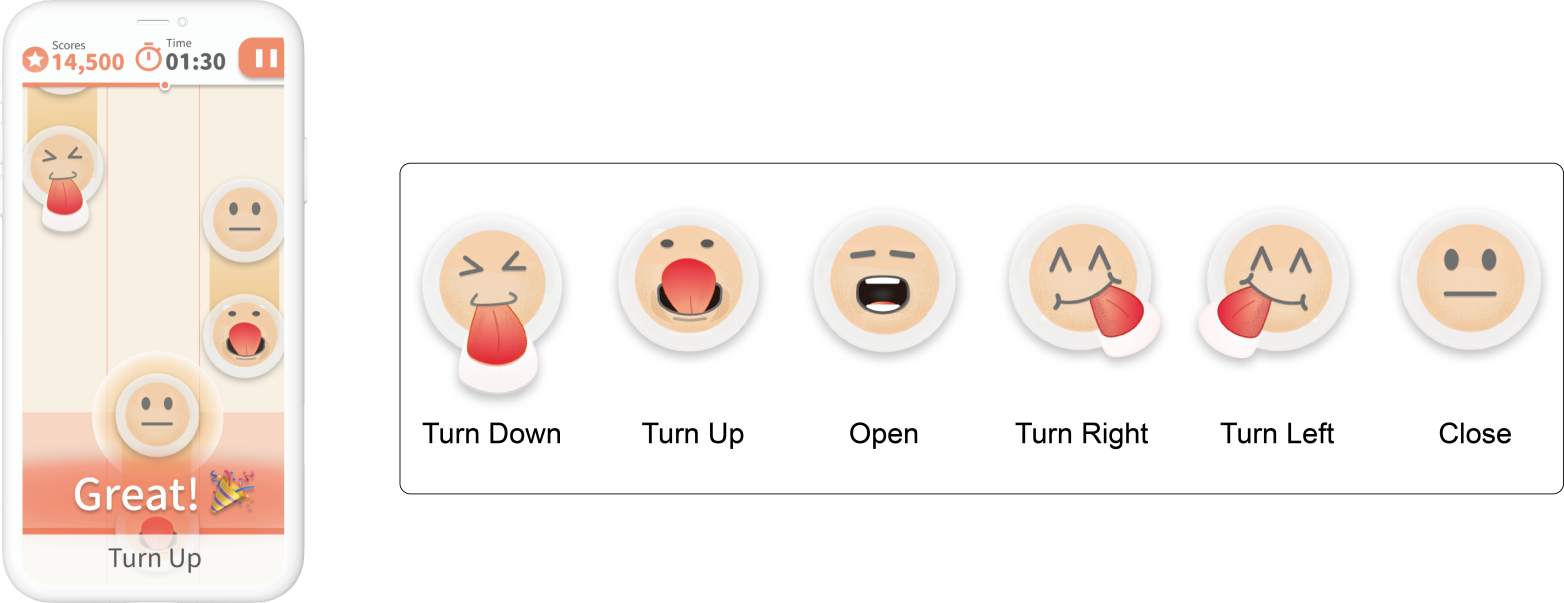
Mobile game interface and the schematic diagram of tongue training actions in the game.

We first photographed 60 participants and removed blurry sections. Teachable Machine version 1.0.1 (Google) was used for initial model training. We imported 200 images for each of the 6 tongue training movements (ie, a total of 1200 training images) to verify that the different movements could be accurately recognized by the camera. The recognition accuracy rates for up, down, left, right, close, and open were 100%, 82%, 96%, 83%, 90%, and 97%, respectively. The trained model was output in the Keras vesion 2.2.0 (Oneiros) and Tensorflow Lite version 1.9 (Google Brain Team) formats. We used the Unity Beats Detection module of Unity Engine version 2020.3.48f1 (Unity Technologies) to analyze the rhythm tempo, and we also used lively and fast-paced music, which began playing at the beginning of the game. In the program design, we generated training levels using the training movements to give the participants a gaming experience, thereby producing an exergame (Figure 1).

Furthermore, to ensure proper hygiene, image recognition methods that did not require invasion into the oral cavity were used. Images of tongue movements were detected, and suitable feedback and reward mechanisms were also provided. We set up 3 levels with varying speeds (slow, medium, and fast) and skill difficulty requirements to test the mental flow state of the participants under different conditions.

A smartphone was used to record the participant’s heart rate (Garmin heart rate chest belt) and mental flow score. To set up the Garmin HRM-PRO activity tracker belt, we created an anonymous user account with the manufacturer that was not associated with any of the patients. Before beginning the game, the participants were separated into the 3 training modes (M1: slow speed; M2: medium speed; or M3: fast speed) by a Latin square method. They then filled out their personal information, read and reviewed their rights and interests, and gave their informed consent. They then put on the Garmin HRM-PRO belt and were asked to rest quietly for 5 minutes before beginning the game tasks. They were given instructions and allowed to practice for 3 minutes, and then played in assigned modes of different difficulties. Subsequently, they rested for 3 minutes and then played again. After each game, they were given a questionnaire (including the FSS2, FD, the NASA-TLX, the UEQ, pragmatic quality, hedonic quality, and NPS), and at the end, an interview was conducted. Using physiological and psychological signals, we explored and analyzed whether the tongue muscle training app could produce a positive flow experience.

**Figure 2.**
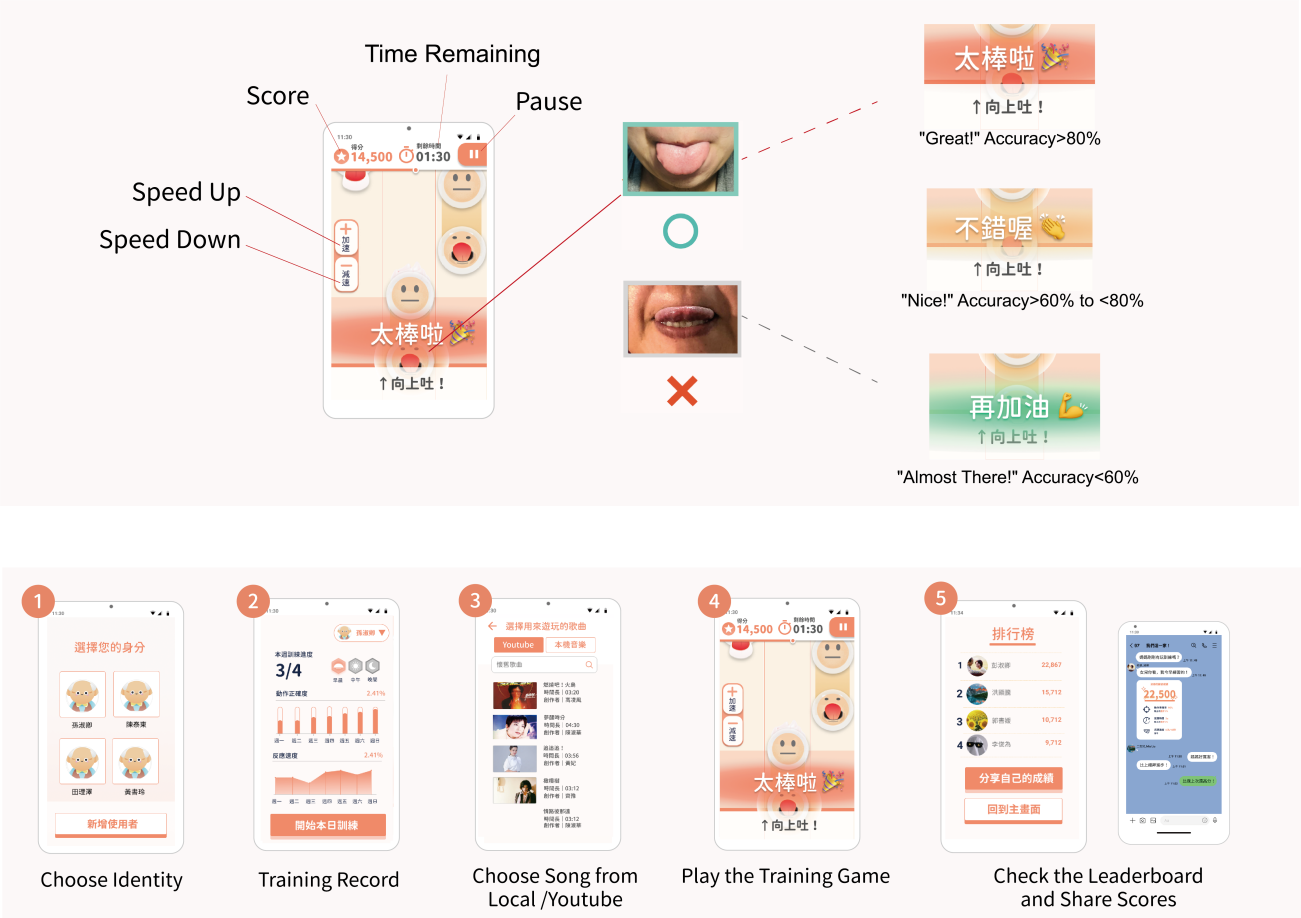
Development of the mobile app interface, the feedback diagram, and the game step flow chart.

After logging in to the daily training page, the homepage was shown. The subject could then select previous records or enter the song menu to choose their favorite music. By tapping the play icon, they could begin training, and after completion, the screen showed the resulting score and their ranking among friends and relatives, which could be shared on social networks (Figure 2). By emulating the correct tongue movements, the system presented encouraging sounds and words such as great, nice, and almost there to the subject. This study explored whether psychological and physiological signals influenced each other, thereby leading to optimal flow states in participants, with the purpose of maintaining the training motivation of middle-aged and older adults. The study integrated big data that could also be used for future smart oral-medical research and analysis.

### Phase 2: Feasibility Testing

This study included a single-arm, unblinded evaluation of the feasibility and acceptability of the tongue app, in preparation for a planned randomized pilot trial. All participants in the feasibility study were given free access to the tongue app. Participants’ use of the app was passively monitored for the pilot trial.

In the early stages, Figma version 95.7 (Figma Inc) was used to create a preliminary model, and the Wizard of Oz method was used to conduct a usability test on 5 middle-aged and older adults aged 55-70 years. After iterative improvements, the final version of the gaming app was developed. We officially started testing on October 5, 2021. All testers were either recruited from Facebook or from friends and relatives of the researchers.

A Google Form published on Facebook served as our web-based survey platform. Recruitment remained open until the prespecified sample size of 32 (7 males, 21.9%; 25 females, 78.1%) healthy middle-aged and older participants was met. We confirmed the eligibility of the test takers based on a questionnaire (open between September 30, 2021, and October 4, 2021) and ensured that participants consented to the feasibility trial. Note that there was a 100% (32/32) response rate for the baseline questionnaire and that this was the denominator used for most of the analyses.

All participants filled in the informed consent form and swallowing function test form (EAT-10). People with specific illnesses or health problems (eg, those with pre-existing dysphagia or cancer) were excluded from this study, and only settings dedicated to home or informal care for older adults were included. In this study, the application was only functional on Android phones, tablets, and computers. We aim to upload the app to the Google Play Store in the near future.

### Ethical Considerations

Taipei Medical University-Joint Institutional Review Board approved this study (N202109022), which was planned, carried out, analyzed, and interpreted independently of any industrial partners. Details about procedures, potential risks, confidentiality measures, data storage protocols, and benefits were shared with all eligible participants. Signed informed consent was collected from each participant. To protect confidentiality and ensure anonymity, participant names were replaced with unique identification codes. Digital data were securely stored on encrypted and password-protected systems. Participants in this study received US $15 as a transport subsidy. The specifics of the compensation were clearly communicated during the consent process.

### Data Collection

This study was based on the baseline model game experience. After answering the questionnaire items regarding demographic background, the participants began to play. During the experiment, each participant played the game for 3 minutes with a song, playing 3 times in each of 3 difficulty levels (M1: slow; M2: medium; M3: fast). Physiological signals (heart rate, low frequency HRV, high frequency HRV, LF/HF ratio, lnRMSSD, RMSSD, NN50, PNN50, and SDNN) were collected by the Garmin HRM-PRO. After the game, participants filled out the rest of the questionnaire, which comprised the following scales: FD, FSS2, NPS, UEQ, System Usability Scale, and NASA-TLX. In the questionnaire, there were measures for the challenge level of each exercise and measures of the skill level of the participant.

Subtracting the challenge score from the skill score and dividing it by 4 resulted in the FD, a score that fell between 1 and −1. A score of 0 meant that the skill and challenge levels were balanced, which was considered to be the state of flow. If a score was between 0 and 1, the subject was classified as bored (ie, the person’s skill levels were higher than the challenge level). If a score was between −1 and 0, the person was classified as anxious. We integrated the questionnaire data and physiological data, which were downloaded from Elitehrv’s paid platform (version 5.5.8, Elite HRV Inc) and organized in an Excel (version 1808, Microsoft) spreadsheet.

### Statistical Analysis

In this study, SPSS version 30 (IBM Corp) was used as the main analysis software, and ANOVA tests were used to analyze whether differences among individual FDs in the M1 (slow), M2 (medium), and M3 (fast) levels were significant. Paired 2-tailed sample *t* tests were used to further analyze whether the training in the 3 levels affected the learning results in terms of operability. In addition, we also investigated whether there were differences between sexes, and the Pearson correlation coefficient was used to explore the relationship between the FSS2 scores and physiological signals. Exploratory factor analysis was used to extract the two factors (operation and immersion), and cluster analysis and k-means clustering were also used to divide the population into 3 groups based on speed, before reperforming the Pearson analysis.

## Results

The reliability of the FSS2 questionnaire, as measured by the Cronbach α, was 0.802. An exploratory factor analysis was conducted on questions 1 to 9 of the FSS2 questionnaire, yielding a *χ*²_36_ of 448.478 (*P*<.001; Kaiser-Meyer-Olkin=0.757). The FSS2 questionnaire was divided into two factors that confirmed the reliability of the structure: the FSS2-01 (FSS2 items 5, 7, 8, and 9) for immersion and FSS2-02 (FSS2 items 1, 2, 3, 4, and 6) for operation. An ANOVA test found that the *F* values were >0.05, indicating that the M1, M2, and M3 training modes all significantly affected the FSS2-02 (*P=*.001), NASA-TLX (*P*<.001), and FD (*P*<.001).

Within these 3 modes, there were significant differences in load level, flow, and FD (*P*<.001; Table 2). A Scheffe post hoc test confirmed that there was a significant difference in the NASA-TLX score between the M1 and M3 modes and between the M2 and M3 modes. There was a significant difference in the FSS2-02 score between the M1 and M3 modes. For FD, there were significant differences between the M1 and M2 modes, as well as the M1 and M3 modes (Multimedia Appendix 1).

**Table 2.** Relationship between the 3 training modes (M1, M2, and M3) and flow outcomes.

Variable	Sum of squares	Mean square	*F* test (*df*)	*P* value^a^
NASA-TLX^b^	1116823.000	558411.500	7.887 (2, 93)	.001
FSS2-02^c^	147.896	73.948	8.542 (2, 93)	<.001
Flow distance	27513.021	13756.510	12.198 (2, 93)	<.001

a The α level was .01 (2-tailed).

b NASA-TLX: National Aeronautics and Space Administration Task Load Index.

c FSS2-02: Flow State Scale 2, items 1, 2, 3, 4, and 6.

In order to determine the degree of influence of the FSS2-02 score on flow between each training mode pair, paired 2-tailed sample *t* tests were used. Significant differences were found among the 3 modes, indicating that participants were in the state of operation and demonstrating that the task design of this study was effective (Table 3). However, for the FSS2-01 questionnaire, only M1 and M3 exhibited a significant difference in immersion (*P=*.03). This could indicate that, in the M2 mode, the ease or difficulty of tasks was biased toward the M1 or M3 modes ().

**Table 3.** Valid operations by participants in the 3 modes (M1, M2, and M3).

Mode^a^ comparisons	Mean difference (SD)	*t* test (*df*)		*P* value^b^
FSS2-02^c^ (operation)				
M1 to M2	–1.71875 (2.61798)	–3.714 (31)		.001^d^
M2 to M3	–1.31250 (2.57077)	–2.888 (31)		.007^d^
M1 to M3	–3.03125 (4.18511)	–4.097 (31)		<.001^d^
FSS2-01^e^ (immersion)				
M1 to M2	0.40625 (2.56351)	0.896 (31)		.38^f^
M2 to M3	0.68750 (3.71950)	1.046 (31)		.30^f^
M1 to M3	1.09375 (2.64404)	2.340 (31)		.03^f^

a Mode: training modes slow (M1), medium (M2), and fast (M3).

b Significance was determined using a one-way ANOVA.

c FSS2-02: Flow State Scale 2, items 1, 2, 3, 4, and 6.

d The α level was .01 (2-tailed).

e FSS2-01: Flow State Scale 2, items 5, 7, 8, and 9.

f The α level was .05 (2-tailed).

In addition, the results of the *t* tests (n=32) showed a significant difference in heart rate between men and women in the M1 mode (*t*_28_=3.697; *P=*.001) and a significant difference in load level indicated by the NASA-TLX (*t*_30_=2.276; *P*=.03). For the M2 mode, only the heart rate was significantly different between men and women (*t*_30_=4.791; *P*<.001). Finally, there were significant differences in flow based on the FSS2-01 (*t*_28_=5.431; *P*<.001), FSS2-02 (*t*_29_=3.379; *P=*.002), FD (*t*_30_=2.043; *P*=.05), as well as a significant difference in heart rate (*t*_30_=3.693; *P*=.001) between men and women in the M3 mode (Table 4).

**Table 4.** Relationship between sex and physiological signals in the 3 mobile game modes (M1, M2, and M3).

Measurements	*F* test (*df*)	*P* value	*t* test (*df*)		*P* value
Slow training mode (M1)					
Heart rate^a^	5.228 (30, 27.5)	.03	3.697 (28)		.001^b^
NASA-TLX^c^	1.720 (30, 7.9)	.20	2.276 (30)		.03^d^
Medium training mode (M2)					
Heart rate	1.233 (30, 7.8)	.28	4.791 (30)		<.001^b^
Fast training mode (M3)					
FSS2-01^e^	5.496 (30, 28)	.03	5.431 (28)		<.001^b^
FSS2-02^f^	5.921(30, 29)	.02	3.379 (29)		.002^b^
Flow distance	0.601 (30, 8.5)	.44	2.043 (30)		.050^d^
Heart rate	0.832 (30, 8.3)	.37	3.693 (30)		.001^b^

a Measured in beats per minute.

b The α level was .01 (2-tailed).

c NASA-TLX: National Aeronautics and Space Administration Task Load Index.

d The α level was .05 (2-tailed).

e FSS2-01: Flow State Scale 2, items 5, 7, 8, and 9.

f FSS2-02: Flow State Scale 2, items 1, 2, 3, 4, and 6.

A Pearson correlation for physiological and psychological signals was performed for each training task mode and we found that the FSS2-01 score was significantly related to age (*r*=0.39; *P*<.001), pragmatic quality (*r*=0.21; *P=*.02), NASA-TLX score (*r*=–0.30; *P*=.003), FSS2-02 score (*r*=0.32; *P=*.001), and FD (*r*=0.33; *P*=.001). The FSS2-02 score was significantly correlated with age (*r*=0.34;* P=*.001), FSS2-01 score (*r*=0.32; *P=*.001), and FD (*r*=–0.34; *P=*.001). In addition, the FSS2-02 score was nonsignificantly correlated with the LF/HF ratio (*r*=–0.20; *P=*.054).

**Figure 3.**
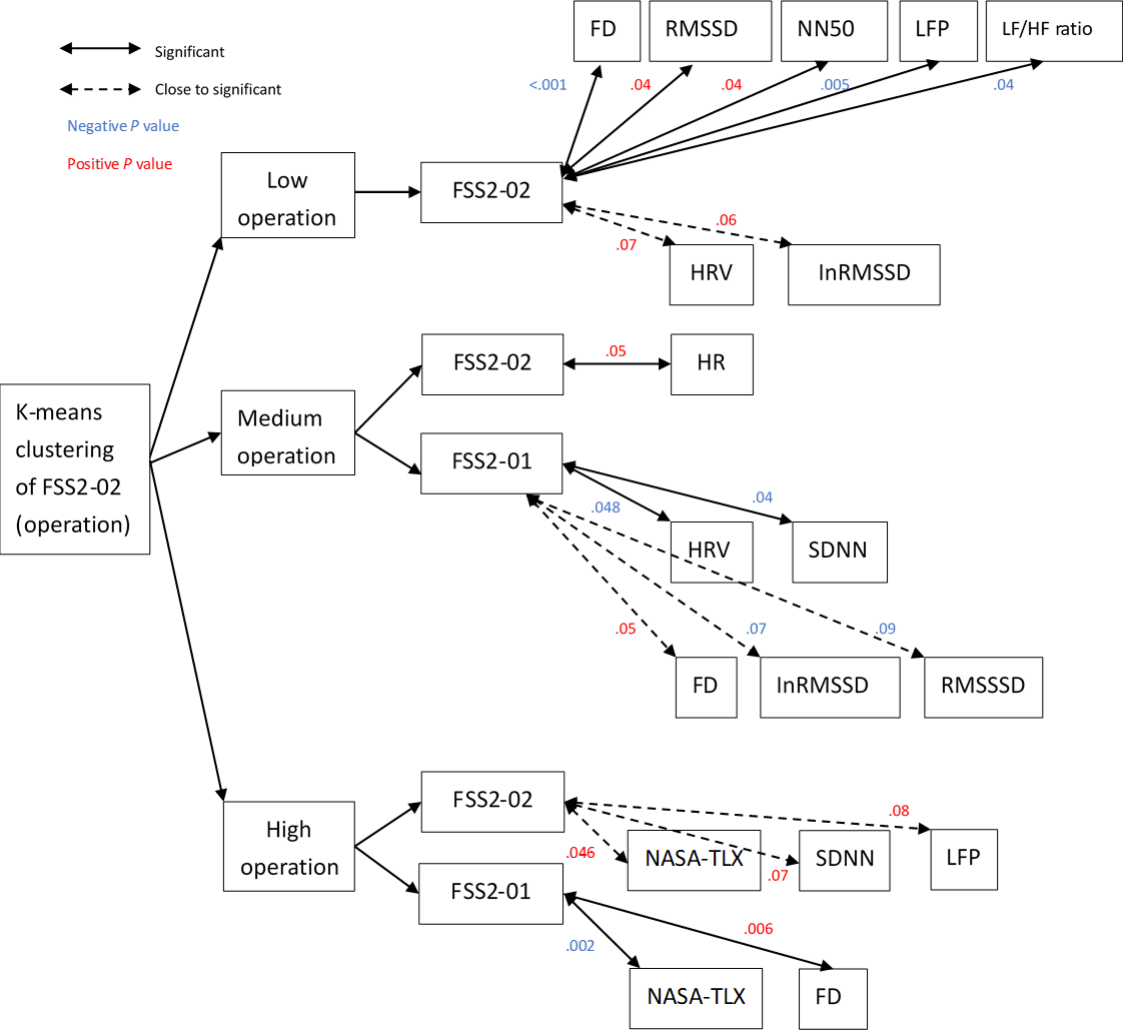
K-means clustering of FSS2-02 divided the relationship between mental flow and various physiological signals into 3 groups. Positive *P* values indicate a positive correlation, and negative *P* values indicate a negative correlation. FD: flow distance; FSS2-01: Flow State Scale 2, items 5, 7, 8, and 9; FSS2-02: Flow State Scale 2, items 1, 2, 3, 4, and 6; HR: heart rate; HRV: heart rate variability; LF/HF: low frequency to high frequency; LFP: low frequency power; lnRMSSD: natural logarithm of the root mean square of the successive differences; NN50: number of pairs of successive NN intervals that differ by more than 50 milliseconds; RMSSD: root mean square of the successive differences; SDNN: SD of the NN interval.

In order to explore whether certain training groups correlated with physiological and psychological signals, we divided the data into 3 groups through k-means clustering, partitioning the FSS2-02 scores into low, medium, and high operation groups. Correlation analysis between the 3 groups and physiological signals found that the FSS2-02 score for the low operation group was significantly correlated to FD (*r*=–0.79; *P<*.001), RMSSD (*r*=0.46; *P=*.04), NN50 (*r*=0.46; *P=*.04), LFP (*r*=–0.60; *P=*.005), and LF/HF ratio (*r*=–0.47; *P=*.04). There was a low correlation between the FSS2-02 score and HRV (*r*=0.42; *P=*.07) and lnRMSSD (*r*=0.43; *P=*.06). In the medium operation group, the FSS2-02 score correlated with heart rate (*r*=0.41; *P=*.050), while the FSS2-01 score correlated with age (*r*=0.58; *P=*.003), pragmatic quality (*r*=0.56; *P=*.005), UEQ score (*r*=0.48; *P=*.02), HRV (*r*=–0.41; *P=*.048), and the SDNN (*r*=–0.43; *P=*.04) and nonsignificantly correlated to hedonic quality (*r*=0.37; *P=*.07), FD (*r*=0.40; *P=*.053), lnRMSSD (*r*=–0.38; *P=*.07), and RMSSD (*r*=–0.35; *P=*.09). In the high operation group, the FSS2-02 score was correlated with the NASA-TLX score (*r*=0.28; *P=*.046) and was nonsignificantly correlated with SDNN (*r*=0.07; *P=*.07) and LFP (*r*=0.24; *P=*.08), while the FSS2-01 score was correlated with the NASA-TLX score (*r*=–0.41; *P*=.002) and FD (*r*=0.38; *P=*.006; Figure 3).

**Figure 4.**
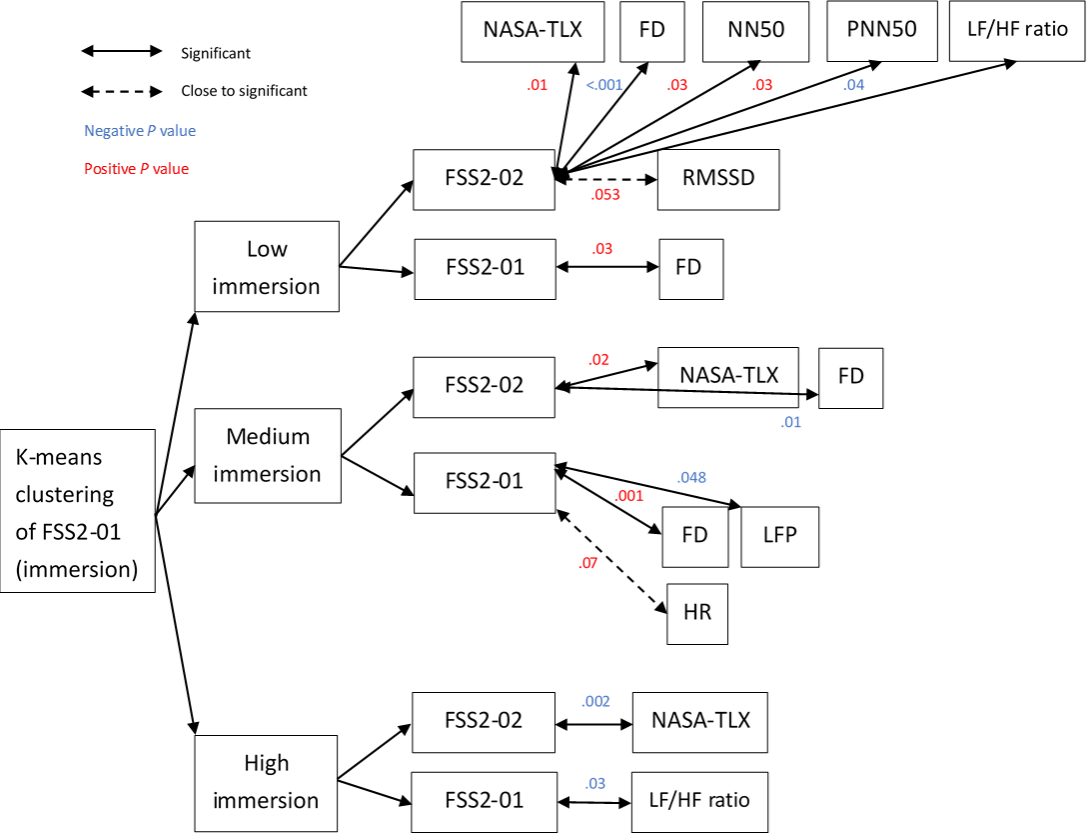
K-means clustering of FSS2-01 divided the relationship between heart flow and various physiological signals into 3 groups. Positive *P* values indicate a positive correlation, and negative *P* values indicate a negative correlation. FD: flow distance FSS2-01: Flow State Scale 2, items 5, 7, 8, and 9; FSS2-02: Flow State Scale 2, items 1, 2, 3, 4, and 6; HR: heart rate; HRV: heart rate variability; LF/HF: low frequency to high frequency; LFP: low frequency power; NASA-TLX: National Aeronautics and Space Administration Task Load Index; NN50: number of pairs of successive NN intervals that differ by more than 50 milliseconds; PNN50: proportion of NN50 divided by the total number of NN intervals; RMSSD: root mean square of the successive differences.

In addition, the research data for the FSS2-01 was divided into low, medium, and high immersion groups through k-means. Correlation analysis with the physiological signals found that the FSS2-02 score in the low immersion group was related to the NASA-TLX score (*r*=0.46; *P=*.009), FD (*r*=–0.69; *P*<.001), NN50 (*r*=0.39; *P=*.03), PNN50 (*r*=0.38; *P=*.03), and LF/HF ratio (*r*=–0.37; *P=*.04), while the FSS2-02 score had a low correlation with age (*r*=0.33; *P=*.07) and RMSSD (*r*=0.35; *P=*.053), and the FSS2-01 score had a positive correlation with FD (*r*=0.39; *P=*.03). On the other hand, in the medium immersion group, the FSS2-02 score was correlated with the NASA-TLX score (*r*=0.39; *P=*.02) and FD (*r*=–0.42; *P*=.01), while the FSS2-01 score was correlated with hedonic quality (*r*=0.35; *P=*.04), UEQ score (*r*=0.36; *P=*.04), FD (*r*=0.53; *P=*.001), and LFP (*r*=–0.34; *P*=.048) and was nonsignificantly correlated with heart rate (*r*=0.31; *P*=.07). Finally, in the high immersion group, it was found that the FSS2-01 score exhibited a negative correlation with LF/HF ratio (*r*=–0.38; *P*=.03) and a low correlation with pragmatic quality (*r*=0.31; *P*=.08), and there was a negative correlation between the FSS2-02 and NASA-TLX scores (*r*=–0.51; *P*=.002; Figure 4).

## Discussion

### Principal Findings

We successfully identified 6 different methods for tongue training and developed a mobile game app for this purpose, aiming for users to experience flow through the training of specialized muscles (ie, the tongue). Most studies focuses on psychological and physiological signals (shown in Figure 3 and Figure 4), and this study emphasized 12 of these indicators (ie, NASA-TLX score, FD, heart rate, HRV, lnRMSSD, RMSSD, NN50, PNN50, SDNN, LFP, high frequency power, and LF/HF ratio). Among the psychological and physiological signals, flow was measured using FD, SDNN, RMSSD, NN50, HRV, and LFP. The LF/HF ratio was also used, which indicated if the findings were related to FD and if these factors generated flow. We found that our participants had effective learning operations and immersive experiences during the tasks and achieved optimal flow, confirming the effectiveness of the app. Importantly, flow generated during gameplay is known to be affected by age and sex, which in turn affects physiological signals, such as HRV [37].

In our mobile health app, interactive games were designed to train tongue muscles, which could be conducted anytime and anywhere, reducing the burden of conventional training. These games can also increase task repetition [55-57], which influences learning effectiveness [58,59]. The tasks were designed with 3 different levels of difficulty. Research has confirmed that different game designs can help participants to improve operability and that introducing gamification into a task mechanism can intensify the balance of physiological and psychological signals [18]. When the balance between challenge and skill is achieved, flow optimization can be achieved [60]. Our findings demonstrate that the participants were immersed in training during the game, confirming the effectiveness of the tongue training app. By changing its speed and rhythm, we could modulate a user’s flow experience. The tempo of the music was linked to the speed at which the tongue instruction icons moved down the smartphone screen, and training levels were generated. Thus, the M3 mode tasks were difficult and the most fast-paced. The participants had to sync the speed of the tongue training motions to the rhythm of the music. This study combined image recognition and music rhythm stimulation to explore and analyze complex physiological and psychological signals. The results show that such methods could generate an ideal flow experience.

### Strengths and Weaknesses of the Study

Our research has successfully simplified complicated rehabilitation procedures, using a simple game app combined with audio effects to facilitate tongue training. To verify the effectiveness of the mobile app games, the FSS2 questionnaire for the game was divided into two groups by exploratory factor analyses: operation and immersion. An analysis of the results for the tasks performed at different speeds revealed that training outcomes were highly related to FD. This demonstrates that through this tongue training app, we can both facilitate tongue muscle rehabilitation and generate flow in users. Flow includes experiencing complete concentration, the desire to challenge oneself, and the generation of feedback reward mechanisms [61], supporting that physiological signals are affected by task stimulation [62].

The image recognition system had certain limitations. Data collection during experiments was also a time-consuming process. In our study, the participant had to wear a heart rate detector during the test, which may have caused some inconvenience and led to minor variations in heart rhythm or physiological signals. Second, research has found that there are indeed differences in flow and physiological signals between men and women, indicating differences regarding the influence of personal characteristics and environmental factors [37]. Under the same difficulty settings, results showed that there were fewer psychological signals in the M3 training mode. In the future, we can appropriately adjust the task difficulty of the M3 mode to achieve optimal training. In addition, despite the small sample size and the skewed sex ratio of the participants in this study, our image recognition system achieved a high level of accuracy, paving the way for scaling up the recruitment of participants in future experiments.

The model of this complete mobile app game was trained using a limited dataset and thus only recognized the mouths and tongues of Asian individuals. Therefore, if the mobile app was applied to non-Asian individuals, it may not perform well, leading to inaccurate experiments. Moreover, these mobile app games were only used by middle-aged and older adults, and the findings may not apply to users in other age groups. In the future, we aim to collect more diverse data to improve the accuracy and breadth of the platform.

### Implications and Future Research

We expect to include the addition of a 2-player competition mode to the interface to enable not only interactions with family or friends but also to include a wider range of advanced training functions. The interface for middle-aged and older adults could be optimized with more options, such as realistic characters and diverse oral and tongue exercises, to enhance the experience of the app. The interface only allowed users to change basic settings such as age, music speed, and mode. Since this study was a pilot study, there were no experiment cases without accompanying soundtracks (ie, during the trial, there were corresponding soundtracks in all 3 modes).

In this study, the generation of a soundtrack required an internet connection. An offline training mode is planned for subsequent iterations of the app so that full training can be achieved anytime and anywhere, even in places with poor signal reception, such as rural areas, elevators, or basements. Furthermore, the soundtrack could be adjusted based on pitch or volume, or it could be muted in future experiments. In this study, our main goal was to provide either an alternative to clinical rehabilitation or a more relaxed training setting for healthy people. When used in actual clinical settings, complying with medical standards and regulations could require the demonstration of efficacy across multiple conditions. For example, the app may be further modified to include oral training. Moreover, the development of the image recognition system was susceptible to slight errors depending on variations in ambient lighting. In the future, we hope to enhance the accuracy of the image recognition system and collect more lighting data to reduce errors under different scenes and levels of light.

### Conclusions

Despite the many ways to train tongue muscles, most studies have been concerned with the treatment and rehabilitation of patients with oral cavity diseases. In addition, equipment expenses or a lack of continuous professional medical services are associated with declines in patient compliance for rehabilitation. There are few gamified apps targeting health care professions and education and even fewer considering factors that may increase efficacy. Following the completion of the pilot trial and subsequent analysis, the participant cohort could be categorized into two groups based on their physiological and psychological signals: operation and immersion. Furthermore, tongue muscle training using our app could produce a superior flow experience for users. This was determined using several variables including FD, SDNN, RMSSD, NN50, HRV, LFP, and LF/HF ratio and could be repeated in different training groups, which confirmed the effectiveness of this app. Compared to strength training and full-body movement training, our method was more engaging and produced a better flow experience. We hope that the optimal flow generated by this app will encourage users to train independently and mitigate or prevent dysphagia. This study strengthens the potential for incorporating game training into mobile health.

## Supplementary material

10.2196/53045 Multimedia Appendix 1 Multiple comparisons from Scheffe test.

10.2196/53045 Checklist 1 Reporting a pilot and feasibility trial checklist.

## Abbreviations

EAT-10: 10-item Eating Assessment Tool
FD: flow distance
FSS2: Flow State Scale 2
FSS2-01: Flow State Scale 2, items 5, 7, 8, and 9
FSS2-02: Flow State Scale 2, items 1, 2, 3, 4, and 6
HRV: heart rate variability
LF/HF: low frequency to high frequency
LFP: low frequency power
lnRMSSD: natural logarithm of the root mean square of successive differences
NASA-TLX: National Aeronautics and Space Administration Task Load Index
NN50: number of pairs of successive NN intervals that differ by more than 50 milliseconds
NPS: net promoter score
PNN50: proportion of NN50 divided by the total number of NN intervals
RMSSD: root mean square of successive differences
SDNN: SD of the NN interval
UEQ: User Experience Questionnaire

## References

[1] Busch PA, Hausvik GI, Ropstad OK, Pettersen D (2021). Smartphone usage among older adults. Comput Hum Behav.

[2] Turel O, Serenko A (2012). The benefits and dangers of enjoyment with social networking websites. Eur J Inf Syst.

[3] Lee JA, Nguyen AL, Berg J (2014). Attitudes and preferences on the use of mobile health technology and health games for self-management: interviews with older adults on anticoagulation therapy. JMIR Mhealth Uhealth.

[4] Foster M, Xiong W, Quintiliani L, Hartmann CW, Gaehde S (2022). Preferences of older adult veterans with heart failure for engaging with mobile health technology to support self-care: qualitative interview study among patients with heart failure and content analysis. JMIR Form Res.

[5] Sheikhany AR, Hady AFA, Farag S (2019). Oropharyngeal dysphagia profile in early versus late stage dementia. Egypt J Otolaryngol.

[6] Singh S, Hamdy S (2006). Dysphagia in stroke patients. Postgrad Med J.

[7] Tjaden K (2008). Speech and swallowing in Parkinson’s disease. Top Geriatr Rehabil.

[8] Manabe T, Teramoto S, Tamiya N, Okochi J, Hizawa N (2015). Risk factors for aspiration pneumonia in older adults. PLoS ONE.

[9] Ekberg O, Hamdy S, Woisard V, Wuttge-Hannig A, Ortega P (2002). Social and psychological burden of dysphagia: its impact on diagnosis and treatment. Dysphagia.

[10] Takeuchi N, Sawada N, Ekuni D, Morita M (2021). Oral diadochokinesis is related to decline in swallowing function among community-dwelling Japanese elderly: a cross-sectional study. Aging Clin Exp Res.

[11] Robbins J, Kays SA, Gangnon RE (2007). The effects of lingual exercise in stroke patients with dysphagia. Arch Phys Med Rehabil.

[12] Antunes EB, Lunet N (2012). Effects of the head lift exercise on the swallow function: a systematic review. Gerodontology.

[13] Shaker R, Kern M, Bardan E (1997). Augmentation of deglutitive upper esophageal sphincter opening in the elderly by exercise. Am J Physiol.

[14] Fukuoka T, Ono T, Hori K, Kariyasu M (2022). Effects of tongue-strengthening exercise on tongue strength and effortful swallowing pressure in young healthy adults: a pilot study. J Speech Lang Hear Res.

[15] Carnaby-Mann GD, Crary MA (2010). McNeill dysphagia therapy program: a case-control study. Arch Phys Med Rehabil.

[16] Carnaby-Mann G, Crary MA, Schmalfuss I, Amdur R (2012). “Pharyngocise”: randomized controlled trial of preventative exercises to maintain muscle structure and swallowing function during head-and-neck chemoradiotherapy. Int J Radiat Oncol Biol Phys.

[17] Miller N, Noble E, Jones D, Burn D (2006). Hard to swallow: dysphagia in Parkinson’s disease. Age Ageing.

[18] Kothari M, Svensson P, Jensen J (2013). Training-induced cortical plasticity compared between three tongue-training paradigms. Neuroscience.

[19] Monteblanco Cavalcante M, Fraga I, Dalbosco B (2021). Exergame training-induced neuroplasticity and cognitive improvement in institutionalized older adults: a preliminary investigation. Physiol Behav.

[20] Hou HY, Li HJ (2022). Effects of exergame and video game training on cognitive and physical function in older adults: a randomized controlled trial. Appl Ergon.

[21] Chuang CS, Chen YW, Zeng BY (2022). Effects of modern technology (exergame and virtual reality) assisted rehabilitation vs conventional rehabilitation in patients with Parkinson’s disease: a network meta-analysis of randomised controlled trials. Physiotherapy.

[22] Huang K, Zhao Y, He R (2022). Exergame-based exercise training for depressive symptoms in adults: a systematic review and meta-analysis. Psychol Sport Exerc.

[23] Huang HC, Wong MK, Lu J, Huang WF, Teng CI (2017). Can using exergames improve physical fitness? A 12-week randomized controlled trial. Comput Hum Behav.

[24] Jackson SA, Eklund RC (2002). Assessing flow in physical activity: the flow state scale–2 and dispositional flow scale–2. J Sport Exerc Psychol.

[25] Geng D, Yang K, Fu Z, Zhang Y, Wang C, An H (2022). Circadian stage-dependent and stimulation duration effects of transcutaneous auricular vagus nerve stimulation on heart rate variability. PLoS ONE.

[26] Li D, Gao W (2021). Physiological state assessment and prediction based on multi-sensor fusion in body area network. Biomed Signal Process Control.

[27] Liu Z, Wei W, Fu W (2021). Amplified acquisition of physiological signal in human body communication. J Internet Technol.

[28] Collins T, Woolley SI, Oniani S (2019). Version reporting and assessment approaches for new and updated activity and heart rate monitors. Sensors (Basel).

[29] Patel N, Patel S, Mankad SH (2022). Impact of autoencoder based compact representation on emotion detection from audio. J Ambient Intell Humaniz Comput.

[30] Gantayat SS, Lenka S (2021). Study of algorithms and methods on emotion detection from facial expressions: a review from past research. Comm Softw Netw.

[31] Wu J, Zhang Y, Sun S, Li Q, Zhao X (2022). Generalized zero-shot emotion recognition from body gestures. Appl Intell.

[32] Yang K, Wang C, Gu Y (2021). Behavioral and physiological signals-based deep multimodal approach for mobile emotion recognition. IEEE Trans Affect Comput.

[33] Levenson RW, Carstensen LL, Friesen WV, Ekman P (1991). Emotion, physiology, and expression in old age. Psychol Aging.

[34] Levenson RW (2014). The autonomic nervous system and emotion. Emot Rev.

[35] Ludwig M, Hoffmann K, Endler S, Asteroth A, Wiemeyer J (2018). Measurement, prediction, and control of individual heart rate responses to exercise-basics and options for wearable devices. Front Physiol.

[36] Stauss HM (2003). Heart rate variability. Am J Physiol Regul Integr Comp Physiol.

[37] Zhang J (2007). Effect of age and sex on heart rate variability in healthy subjects. J Manipul Physiol Ther.

[38] Bourdillon N, Yazdani S, Vesin JM, Schmitt L, Millet GP (2022). RMSSD is more sensitive to artifacts than frequency-domain parameters: implication in athletes’ monitoring. J Sports Sci Med.

[39] Garg V, Verma S, Connelly KA (2020). Does empagliflozin modulate the autonomic nervous system among individuals with type 2 diabetes and coronary artery disease? The EMPA-HEART CardioLink-6 Holter analysis. Metabol Open.

[40] Von Bargen T, Zientz C, Haux R (2014). Gamification for mHealth - a review of playful mobile healthcare. Stud Health Technol Inform.

[41] Miller AS, Cafazzo JA, Seto E (2016). A game plan: gamification design principles in mHealth applications for chronic disease management. Health Informatics J.

[42] Csikszentmihalyi M (1996). Creativity: Flow and the Psychology of Discovery and Invention.

[43] Csikszentmihalyi M (1990). Flow: The Psychology of Optimal Experience.

[44] Novak TP, Hoffman DL, Yung YF (2000). Measuring the customer experience in online environments: a structural modeling approach. Mktg Sci.

[45] Csikszentmihalyi M, Hunter J (2003). Happiness and creativity. Fut.

[46] Hoffman DL, Novak TP (1996). Marketing in hypermedia computer-mediated environments: conceptual foundations. J Mark.

[47] Novak TP, Hoffman DL, Duhachek A (2003). The influence of goal-directed and experiential activities on online flow experiences. J Consum Psychol.

[48] Tong Y, Liang Y, Spasic I, Hicks Y, Hu H, Liu Y (2022). A data-driven approach for integrating hedonic quality and pragmatic quality in user experience modeling. J Comput Inf Sci Eng.

[49] Atoum I (2023). Measurement of key performance indicators of user experience based on software requirements. Sci Comput Program.

[50] Bouchard MA, Bergeron-Boucher J, Chamberland C, Tremblay S, Jackson PL (2018). Assessing Differences in Emotional Expressivity Between Expert and Non Expert Video Game Players Using Facial Electromyography. Neuroergonomics.

[51] Choi DS, Kim HY, Kim JW (2000). A cognitive and emotional strategy for computer game design. Asia Pac J Inf Syst.

[52] Chen LX, Sun CT (2016). Self-regulation influence on game play flow state. Comput Hum Behav.

[53] Zhang J, Huang Y, Dong Y, Li J, Zhu L, Zhao M (2024). The effect of music tempo on movement flow. Front Psychol.

[54] Hwu YJ, Kuo CT, Chiang QG (2019). Guidance manual for care and guidance of eating and swallowing difficulties. https://www.mohw.gov.tw/dl-58241-7dacaed8-99a1-4053-aa6a-a7f106435fc6.html.

[55] Huo X, Wang J, Ghovanloo M (2007). A wireless tongue-computer interface using stereo differential magnetic field measurement. Annu Int Conf IEEE Eng Med Biol Soc.

[56] Huo X, Wang J, Ghovanloo M (2008). Introduction and preliminary evaluation of the Tongue Drive System: wireless tongue-operated assistive technology for people with little or no upper-limb function. J Rehabil Res Dev.

[57] Huo X, Wang J, Ghovanloo M (2008). A magneto-inductive sensor based wireless tongue-computer interface. IEEE Trans Neural Syst Rehabil Eng.

[58] Hartmann T, Klimmt C (2006). Gender and computer games: exploring females’ dislikes. J Comp Mediated Comm.

[59] Hartman JM (2007). Self-controlled use of a perceived physical assistance device during a balancing task. Percept Mot Skills.

[60] Shin N (2006). Online learner’s ‘flow’ experience: an empirical study. Brit J Educ Tech.

[61] Bressler DM, Bodzin AM (2013). A mixed methods assessment of students’ flow experiences during a mobile augmented reality science game. Comp Assisted Learn.

[62] Martin-Niedecken AL, Schwarz T, Schättin A (2021). Comparing the impact of heart rate-based in-game adaptations in an exergame-based functional high-intensity interval training on training intensity and experience in healthy young adults. Front Psychol.

