# Correction: Tongue Muscle Training App for Middle-Aged and Older Adults Incorporating Flow-Based Gameplay: Design and Feasibility Pilot Study

**DOI:** 10.2196/90410

**Published:** 2026-01-16

**Authors:** Kuan-Chu Su, Ko-Chiu Wu, Kuei-Ru Chou, Chia-Hsu Huang

**Affiliations:** 1 College of Design, National Taipei University of Technology, Taipei, Taiwan; 2 Department of Interaction Design, National Taipei University of Technology, Rm.701-4, Design Building, No.1, Sec.3, Chung-hsiao E. Rd, Taipei, 10608, Taiwan, 886 912-595408, 886 2-87732913; 3 School of Nursing, Taipei Medical University, Taipei, Taiwan

In “Tongue Muscle Training App for Middle-Aged and Older Adults Incorporating Flow-Based Gameplay: Design and Feasibility Pilot Study” [1], the authors made one change.

The institutional affiliation of author KCS has been changed from the following:

Department of Interaction Design, National Taipei University of Technology, Taipei, Taiwan

The affiliation now reads :

College of Design, National Taipei University of Technology, Taipei, Taiwan

The correction will appear in the online version of the paper on the JMIR Publications website, together with the publication of this correction notice. Because this was made after submission to PubMed, PubMed Central, and other full-text repositories, the corrected article has also been resubmitted to those repositories.
