# Supplementary material for: Tongue Muscle Training App for Middle-Aged and Older Adults Incorporating Flow-Based Gameplay: Design and Feasibility Pilot Study
Source: JMIR Serious Games. 2025 Jan 9;13:e53045. doi: 10.2196/53045 (PMC11737528; doi:10.2196/53045)
Supplement: Multimedia Appendix 1 [file games-v13-e53045-s001.pdf]

Multiple Comparisons from *Scheff*

Variable	*P* value
TLXSum	
M1 - M2	.984
M2 - M3	.005*
M1 - M3	.003*
FSS2-02	
M1 - M2	.070
M2 - M3	.209
M1 - M3	.000*
FD	
M1 - M2	.030*
M2 - M3	.088
M1 - M3	.000*

*. The mean difference is significant at the 0.05 level.
