# Supplementary material for: Tongue Muscle Training App for Middle-Aged and Older Adults Incorporating Flow-Based Gameplay: Design and Feasibility Pilot Study
Source: JMIR Serious Games. 2025 Jan 9;13:e53045. doi: 10.2196/53045 (PMC11737528; doi:10.2196/53045)
Supplement: Checklist 1 [file games-v13-e53045-s002.pdf]

Reporting a pilot and feasibility trial checklist

1a: Identification as a pilot or feasibility randomised trial in the title

We referred to CONSORT 2010 of pilot trials for title- and abstract-specific guidelines. This paper has been revised to "Tongue muscle training app for middle-aged and elderly people incorporating mobile gameplay based on flow experience: Design and Feasibility Pilot Study".

1b: Structured summary of pilot trial design, methods, results, and conclusions (for specific guidance see CONSORT abstract extension for pilot trials)

The abstract has been restructured, following the extension of CONSORT for abstracts for reporting pilot trials. We have rewritten the pilot trial design, including our background, objectives, methods, results, and conclusions, to reflect our study aims.

2a: Scientific background and explanation of rationale for future definitive trial, and reasons for randomised pilot trial

Our research is based on the concept of mhealth technology, which aims to assist users in undergoing rehabilitation anytime and anywhere through mobile platforms. mhealth technology can be used for exercise or well-being game monitoring (Lee et al., 2014). For example, it could assist the elderly with self-care maintenance, including education on daily healthcare, frequency of receiving health education, support of medication adherence, dietary restriction support, goal setting for exercises, stress reduction strategies, and prompts for when to call a provider (Foster et al., 2022).

Here, we aim to prevent and mitigate complications related to dysphagia, such as rapid aging, oral disease, weakened oral expression ability, and aspiration pneumonia in the elderly (Sheikhany, Hady, & Farag, 2019; Singh & Hamdy, 2006; Tjaden, 2008; Manabe et. al, 2015). In our review of the literature, we found several studies related to the use of tongue movement for disease prevention, including the 10-item Eating Assessment Tool (EAT-10) to evaluate swallowing function (Takeuchi, Sawada, Ekuni, & Morita, 2021) and dysphagia improvement through language and tongue movements (Robbins et. al, 2005) (Antunes and Lunet, 2012; Shaker et al., 1997) (Fukuoka, Ono, Hori, and Kariyasu, 2022) (G. D. Carnaby-Mann and Crary, 2010; G. Carnaby-Mann et al., 2012; Miller et al., 2006). We have also found that exergame training can offer more benefits for the elderly by not only alleviating symptoms but also improving strength and balance as well as improving overall fitness (Cavalcante et al., 2021) (Hou & Li, 2022) (Chuang et al., 2022) (K. Huang et al., 2022) (H.-C. Huang, Wong, Lu, Huang, & Teng, 2017). Using signal receivers, we can collect physiological signals generated during exercise, thus allowing us to further analyze the training status of users (Jackson & Eklund, 2002) (Geng, D et al., 2022) (Li & Gao, 2021) (Liu, Wei, & Fu, 2021) (Collins et al., 2019) (Patel, Patel, & Mankad, 2022).

Expanding the scope includes biosensors, which recognize audio features (Patel, Patel, & Mankad, 2022), facial expressions (Gantayat & Lenka, 2021), body gestures (Wu et al., 2022), and even touches

on sensitive screens (Yang et al., 2021). Furthermore, biosensors can be useful in emotion detection by monitoring autonomic nervous system (ANS) activity (R. W. Levenson et al., 1991; Robert W. Levenson, 2014).

However, in game mechanics, because physiological signals and psychological signals are complex interactions, they are rarely discussed and studied together. We discovered that game-based exercise can help users to achieve the optimal immersion experience through the Flow process. There are many Flow-related studies on gaming (Von Bargen, Zientz, & Haux, 2014) (Miller, Cafazzo, & Seto, 2014). Flow refers to an immersive experience that balances abilities and challenges and is a process of optimizing challenges and tasks (Csikszentmihalyi, 1997a; Csikszentmihalyi, 1990) (Novak, Hoffman, and Yung, 2000). The eight dimensions defined in this study include the integration of structures, such as clear goals, feedback, challenge matching skills (Shin, 2006), concentration, control, loss of self-awareness, time transitions, and the automatic nature of activities (Csikszentmihalyi, 1997b).

We found that these factors induce Flow in users (Novak, Hoffman, and Duhachek, 2003). If feedback, challenge, and reward mechanisms are generated in the game, we can capture the user's interest. In many related studies, questionnaires are often used to investigate game experience, including PragmaticQuality, HedonicQuality, Net Promoter Score (NPS), and the User Experience Questionnaire (UEQS), which can explore potential market opportunities (Tong, Y et al., 2022; Atoum, 2023). The NASA-TLX (TLXSum) questionnaire, which assesses the degree of perceived workload, is also commonly used to evaluate the effectiveness of gaming tasks (Bouchard et al., 2018).

In addition, the Flow State Scale-2 (FSS-2), Dispositional Flow Scale-2 (DFS-2), Flow Distance (FD), EGameFlow, and Game Experience Questionnaire (GEQ) can effectively measure flow experience (Jackson SA, Eklund RC. 2002) (Choi, Kim, and Kim, 2000). Among them, FD divides experience into three levels to distinguish the current state of experience: anxiety, flow, and boredom (Chen L-X, Sun C-TJCiHB, 2016). Furthermore, studies have combined music and Flow to improve sports training effects (Zhang et al., 2024).

2b: Specific objectives or research questions for pilot trial

Our contribution lies in the experimentation process, where we detect and collect physiological and psychological signals during gameplay. From the concept of flow distance, we discovered that users have flow experience. We cross-referenced physiological and heart rate signals with questionnaires to explore their correlation. By using a specific measurement method, we investigated the complexity of this relationship and found a correlation. Furthermore, the speed of the game affects the flow state. We divided the results into two groups: immersion and operation. These two groups showed different psychological and physiological data related to the flow state.

Due to the above findings, the purpose of this study was to use the tongue training app that we developed to optimize Flow in elderly people while training tongue muscles. Here, we set up three tasks, each at a different speed, and collected a small number of randomized pilot trials. Based on the Flow experience from 32 users, the feasibility of integrating gameplay into tongue muscle training was

evaluated. The game experience provided the elderly with a fun and useful rehabilitation tool. Analysis of patiens with dysphagia may related to more complex variables, this study has healthy people for simplification in the pilot run. We aim to utilize these findings for the next stage of development and evaluation, hoping to alleviate dysphagia-related conditions and enable the elderly to maintain a healthy lifestyle.

3a: Description of pilot trial design (such as parallel, factorial) including allocation ratio

The study was conducted in two phases. In the first phase, we designed and integrated the gameplay into the tongue training app. In the second phase, we evaluated the feasibility and acceptability of the integrated game using a single-arm trial. In the first part, i.e., pilot trial design, nursing experts from Taipei Medical University formulated the training movements used in the game. Valid movements included “up”, “down”, “left”, “right”, “close”, “open”, and other actions. A total of 1,200 photos from 60 subjects were used for model training. Results exceeded expectations, with recognition accuracy reaching 100% for “up”, 82% for “down”, 96% for “left”, 83% for “right”, 90% for “close”, and 97% for “open”.

The second part was game development and app design. This study included a single-arm, unblinded evaluation of the feasibility and acceptability of the tongue training app. After filling in personal information and signing informed consent forms, the subjects were given a Garmin HRM-PRO and five minutes to accustom themselves to its weight. After a three-minute tutorial, the trial began, in which users were allowed to select the initial difficulty and play mode. During the game, the user's physiological signals and Flow scores were collected using the Garmin heart rate monitor. The subjects played games with three different difficulties, i.e., M1-M3, in the order of a Latin grid. The trial continued with three-minute breaks between each task. Questionnaires (FSS-2, FD, TLXSum, UEQS, PragmaticQuality, HedonicQuality, and NPS tables) and interviews were conducted at the end of the game.

3b : Important changes to methods after pilot trial commencement (such as eligibility criteria), with reasons

This was not applicable because before the pilot experiment, we conducted the Wizard of Oz Method test on five middle-aged and elderly people aged 55 to 70 years old. The game interface and game method were further designed and refined based on the interview results. We also used a Google Form to conduct a survey on the target audience and did not find any participants who did not meet the eligibility criteria. Thus, there were no important changes to the methods after the pilot trial in our study.

4a: Eligibility criteria for participants

Since we recruited participants through an open Google Form on a Facebook web-based survey platform, we conducted an open questionnaire to ensure that participants were eligible (from

September 30, 2021, to October 4, 2021) and indicated consent to the feasibility trial. Inclusion criteria included healthy middle-aged and elderly people. All subjects filled out the informed consent form and EAT-10 Swallowing Function Checklist. Those with specific diseases or health problems (e.g., those pre-existing dysphagia or cancer patients) were excluded from this study.

4b: Settings and locations where the data were collected

Participants in our experiment were recruited from Facebook or introduced by friends through web-based survey platforms. Recruitment remained open until the prespecified sample size of 32 participants was met. The questionnaire included biological gender, age, the EAT-10 Checklist, dysphagia symptom occurrence, exercise software usage, and experience in training or rehabilitation. Implementation sites included homes or informal care settings for older people.

4c: How participants were identified and consented

Based on data from the 32 participants, we determined that they met the criteria of our experimental goals. The interviewer asked them to fill out an informed consent form to confirm their willingness to participate.

5: The interventions for each group with sufficient details to allow replication, including how and when they were actually administered

Our study is a baseline model game experience. The game time of all subjects was three minutes for each song, in which they underwent three different speed tasks (i.e., M1, M2, and M3) three times each. After filling in the user rights and informed consent, they put on the Garmin HRM-PRO belt and rested for 5 minutes before starting the game. Before the game, each participant was given a three-minute tutorial before being allowed to select the initial mode and difficulty. The Garmin HRM-PRO belt simultaneously collected physiological signals (HR, LF-HRV, HF-HRV, LF/HF, InRMSSD, PNN50, and SDNN). The data were downloaded from the paid platform Elitehrv and organized in a Microsoft Excel spreadsheet. After the game, the subjects were required to fill in quantitative questionnaires, such as Flow Distance, FSS-2, NPS, UEQ-S, SUS, NASA-TLX, and interview results. The process was as follows. After logging into the training screen, music was selected prior to entering the main game screen, and the training game was then played. After the training was completed, the training score and the ranking within the user’s friend circle were displayed on the screen, allowing for sharing and communication on social platforms.

6a: Completely defined prespecified assessments or measurements to address each pilot trial objective specified in 2b, including how and when they were assessed

Before planning and development, we conducted interviews with experts and subjects to understand the goals and objectives of game development. Therefore, we expected that the physiological signals and psychological signals to be measured could be collected based on various questionnaires such as

physiological signal receivers and FSS-2. As we already knew that physiological signals could be obtained in a short period of time (Geng, D et al., 2022), the game time was limited to between 3 to 5 minutes. Knowing that training processes requiring full-body movement can lead to optimal Flow, we aimed to elucidate in this study whether this type of localized muscle training method was more attractive than single-strength training and full-body movement training. In our game, the tasks were designed with three different speeds of difficulty and focused on skill training rather than intensity. First, we published a Google Form on a Facebook web-based survey platform to recruit participants. After confirming that they met our inclusion criteria, including age, health status, biological gender, and various other conditions, we conducted individual testing. In order to collect various physiological signals of the participants, we used a Garmin HRM-PRO belt to collect HR, LF-HRV, HF-HRV, LF/HF, InRMSSD, PNN50, and SDNN signals, among others. After the game, subjects filled in questionnaires related to Flow Distance, FSS-2, NPS, UEQ-S, SUS, and NASA-TLX, among others.

6b: Any changes to pilot trial assessments or measurements after the pilot trial commenced, with reasons

This was not applicable. We restricted the target group from the beginning and pre-excluded all those who did not fit our criteria. Our study was based on only a small number of participants, and the tongue training regimen was formulated by experts. Therefore, no changes to the pilot trial occurred during the testing process.

6c: If applicable, prespecified criteria used to judge whether, or how, to proceed with future definitive trial

Before the pilot experiment, we conducted the Wizard of Oz Method test on five middle-aged and elderly people aged 55 to 70. Based on the interview results, we further designed and refined the game interface and contents. We then began to design the pilot trial study to evaluate the feasibility of the game and confirmed that when using the tongue training app, there was an immersive and operational experience embedded in the resulting physiological and psychological signals. Indeed, our app could induce Flow. Following this study, we will conduct a more definitive trial on the app and expand the number of statistical samples. Further analysis such as efficacy in patient treatment will be conducted.

7a: Rationale for numbers in the pilot trial

Browne reported a general rule of using "at least 30 subjects or greater to estimate a parameter" (Browne R. H., 1995). Kieser and Wassmer applied the 80% UCL approach to the sample size calculation and found that a pilot trial sample size between 20 and 40 would minimize the overall sample size for a main study sample size of 80-250, corresponding to standardized effect sizes of 0.4 and 0.7 (for 90% power based on a standard sample size calculation) (Kieser, M., & Wassmer, G., 1996). Thus, this was our rationale for selecting the total number of participants (i.e., 32) in our study.

7b: When applicable, explanation of any interim analyses and stopping guidelines

Before the pilot study, we conducted the Wizard of Oz Method test on five middle-aged and elderly people aged 55 to 70, using the SUS (System Usability Scale) and UEQ user experience scale. The score was 68 points, which was deemed sufficient by Bangor (2009). We further designed the game interface and the game mechanism based on the interview results. Therefore, we did not interrupt the pilot trial study.

8a: Method used to generate the random allocation sequence

The subjects played games at three different speeds (M1-M3) in the order of the Latin square method.

8b: Type of randomisation(s); details of any restriction (such as blocking and block size)

This trial did not have a control group or experimental group. The pre-experimental design was a single-arm trial without randomization.

9: Mechanism used to implement the random allocation sequence (such as sequentially numbered containers), describing any steps taken to conceal the sequence until interventions were assigned

Participants engaged in tasks at three different speeds (i.e., M1, M2, and M3) according to the Latin grid arrangement (Table 1).

Table 1. Latin square order table

Number	Round 01	Round 02	Round 03
1, 7, 13, 19, 25, 31	M1	M2	M3
2, 8, 14, 20, 26, 32	M1	M3	M2
3, 9, 15, 21, 27	M3	M1	M2
4, 10, 16, 22, 28	M3	M2	M1
5, 11, 17, 23, 29	M2	M3	M1
6, 12, 18, 24, 30	M2	M1	M3

10: Who generated the random allocation sequence, who enrolled participants, and who assigned participants to interventions

In this experiment, we separated participants by the Latin square method. Our team constructed a Google Form on a Facebook web-based survey platform to collect the number of participants in an open recruitment activity. As this trial did not have a control group or experimental group and was a pre-experimental design by a single-arm trial, all of the participants received the intervention/training.

11a-11b: If done, who was blinded after assignment to interventions (for example, participants, care providers, those assessing outcomes) and how. / If relevant, description of the similarity of interventions

The study was an open-label trial; thus, all participants received the same treatment. It was not a blinded study.

12: Methods used to address each pilot trial objective whether qualitative or quantitative

This study used the Statistical Package for the Social Sciences (SPSS) as the main analysis software as well as the Analysis of Variance (ANOVA) to analyze the conditions under three different speeds and techniques, namely modes M1 (slow), M2 (mid), M3 (fast). The significance was determined using paired sample T-tests between individual FDs to analyze whether training in the three modes affected the learning results in terms of operability. We also used the Pearson correlation coefficient to explore the relationship between physiological and psychological signals to elucidate the relationship between FSS-2 and physiological signals. Cluster Analysis and K-Means were also used to divide the data into two groups in order to re-perform the Pearson correlation test.

13a: For each group, the numbers of participants who were approached and/or assessed for eligibility, randomly assigned, received intended treatment, and were assessed for each objective

A usability test was conducted on five middle-aged and elderly people aged 55 to 70 years old. After iterative improvements, the app was refined and finalized. The project officially started on October 5, 2021. All subjects were recruited either from Facebook or through acquaintances of the researchers. Recruitment remained open until the prespecified sample size of 32 participants was met. All subjects were required to fill in the informed consent form and the EAT-10 Checklist. People with specific diseases or health problems (e.g., patients with pre-existing dysphagia or cancer) were removed. There were 7 males and 25 females in our cohort (Figure 1)

Figure 1. Flow chat of Recruitment and Participants

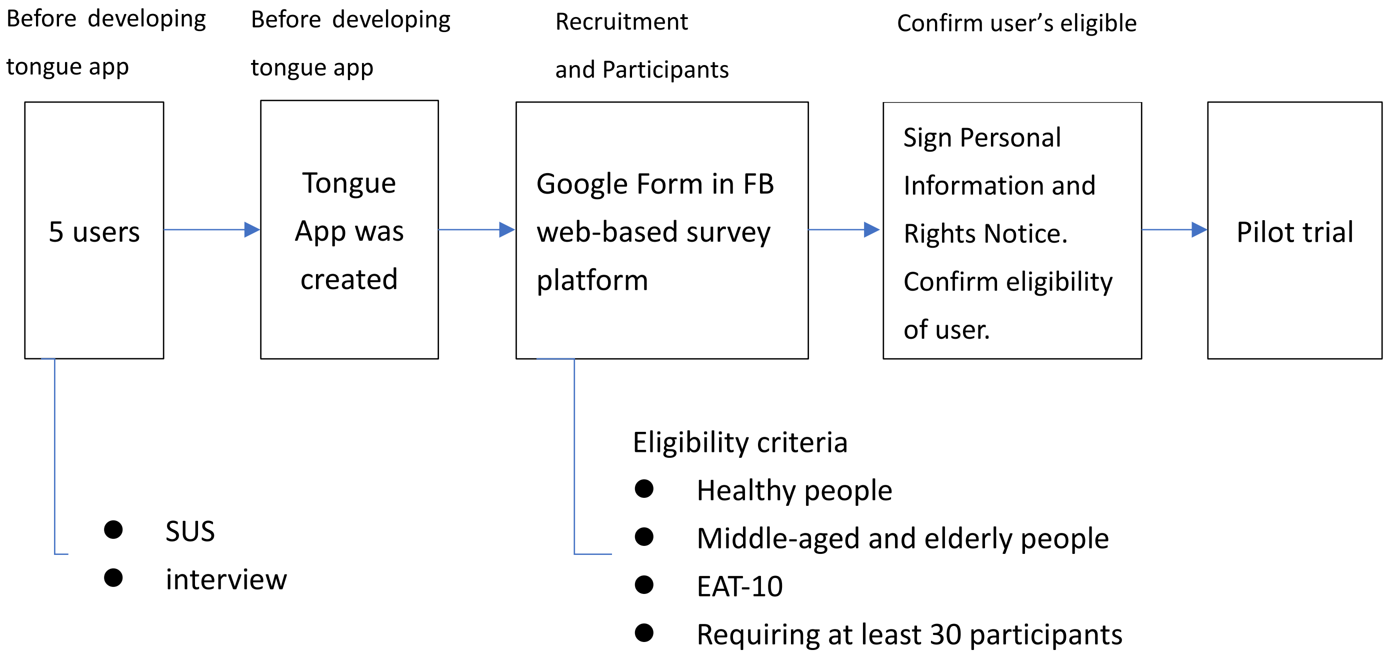

13b: For each group, losses and exclusions after randomisation, together with reasons

This was not applicable. Our experiment included all those who met the criteria for inclusion in this study, such that none were excluded or eliminated based on any post-experiment results. This study was not a study comparing the efficacy of an experimental group with a control group. We developed an app for tongue training and movements and analyzed the results based on the collected physiological and psychological signals.

14a: Dates defining the periods of recruitment and follow-up

The project commenced with a questionnaire on test subject qualification (from September 30, 2021, to October 4, 2021), and the trial officially began on October 5, 2021.

14b: Why the pilot trial ended or was stopped

The pilot trial ended at the conclusion of intended end date. There were no reasons that necessitated the trial to end early.

15: A table showing baseline demographic and clinical characteristics for each group

We mainly conducted experiments on healthy middle-aged and older Asians. People with certain diseases or health problems (e.g., those with pre-existing dysphagia or cancer) or people from non-Asian regions were excluded from this study. This was because such inclusions would involve efficacy, which would have complicated the study results. In addition, the presence of non-Asian subjects may have affected detection accuracy.

16: For each objective, number of participants (denominator) included in each analysis. If relevant, these numbers should be by randomised group

The cohort of test subjects in the experiment was composed of 7 males and 25 females for a total of 32 people (n=32), who could be divided into immersion (n=16) and operation (n=16) groups according to K-means clustering.

17: For each objective, results including expressions of uncertainty (such as 95% confidence interval) for any estimates. If relevant, these results should be by randomised group

We developed three tongue training tasks at different speeds. Flow and physiological signals were measured during gameplay, which were subsequently analyzed. This study recruited 32 participants, meeting the criteria of at least 30 subjects or greater to estimate a parameter (Browne R. H., 1995). The reliability of the FSS2 questionnaire, as measured by Cronbach's Alpha, is 0.802. An exploratory factor analysis (EFA) was conducted on questions 1 to 9 of the FSS2 questionnaire, yielding χ^2 = 448.478, df = 36, P<.001 (KMO = .757). The mental flow questionnaire was divided into two factors: immersion and operation, confirming the reliability of the structure. In the determination of changes

in their physiological and psychological signals for each given task, pearson correlation indicated that changes were primarily related to flow direction. Of the 12 indicators (TLXSum, FD, HR, HRV, lnRmssd, rMSSD, Nn50, PNN50, SDNN, LowFrequencyPower, HighFrequencyPower, LF/HF Ratio), the operation-Low group showed significant changes in 7 indicators (58.3%), operation-Mid in 6 (50%), and operation-High in 3 (25%). Similarly, the Immersion-Low group had changes in 6 indicators (50%), Immersion-Mid in 4 (33.3%), and Immersion-High in 2 (16.7%). SPSS analysis was used to analyze the results of this trial, with a 95% confidence interval for each objective.

18: Results of any other analyses performed that could be used to inform the future definitive trial

According to our experimental results, it was found that the relevant data correlated with FD, SDNN, rMSSSD, Nn50, HRV, LFP, and the LF/HF ratio. M1, M2, and M3 all had a significant impact on the results of the FSS2-02 flow questionnaire (p=0.001), TLXsum (p=0.000), and FD (p=0.000), indicating that for all three tasks, the difference in load, Flow, and Flow distance ($p<0.05$) were statistically significant. Note that in the FSS2-01 flow questionnaire, only results from M1 and M3 were significant for immersion (p=0.026), which may indicate that the difficulty of M2 was less than that of either M1 or M3. These findings are all useful in the design of a definitive trial in the future.

19-19a: All important harms or unintended effects in each group (for specific guidance see CONSORT for harms)/ If relevant, other important unintended consequences

The study was not clinical and could not be used to replace medical treatment. For this trial, an interventional experience lasting less than five minutes was adopted. There were no reports of harm or unintended effects on the participants.

20: Pilot trial limitations, addressing sources of potential bias and remaining uncertainty about feasibility

Image recognition has certain limitations. Data collection during the trial was also a time-consuming process. The subject had to wear a heart rate device during the test period, which may have caused inconvenience and slightly influenced the results of the trial. The process may have also caused effects on heart rhythm or physiological signals.

In addition, the sample size of this study was relatively small, with an especially skewed male-to-female ratio toward females. However, since the accuracy was high, there is no reason to preclude the expansion of the sample size in the future.

The image recognition model in this study was only designed to identify Asians. If we had used non-Asian subjects, the detection accuracy may have been affected. Furthermore, the current targets are healthy middle-aged and elderly people. People with specific diseases or health problems (for example, patients with pre-existing dysphagia or cancer) were restricted from participating in the trials to avoid affecting the results. Future patient outcome studies addressing other ethnicities and ages may be

developed.

21: Generalisability (applicability) of pilot trial methods and findings to future definitive trial and other studies

Each trial collected the physiological signals and Flow state of the user. It was found that the user could either be categorized into an operation or immersion mode, indicating that our task design was effective. The results of our analysis showed that this was related to the Flow distance, indicating that in this app, local muscle training could also enable the user to have a flow experience. The tongue training app developed in this research was based on mobile phone interface operation and usage habits. In the future, it will be available for the public to download and register to accomplish more extensive data collection. The applicability of our pilot trial methods and findings could be useful to inform future, more comprehensive trials as well as other studies.

22: Interpretation consistent with pilot trial objectives and findings, balancing potential benefits and harms, and considering other relevant evidence

The goal of this study was to determine whether it was possible to create the experience of Flow through our tongue training app. This was evaluated using both psychological and physiological signals. The results included FD, SDNN, rMSSSD, Nn50, HRV, and LFP; the LF/HF ratio appears repeatedly in different groups, indicating that the results are related to Flow distance and that these factors generate Flow. This finding is worthy of further exploration.

22a: Implications for progression from pilot to future definitive trial, including any proposed amendments

Currently, our app simply provides a baseline experience for healthy elderly people. In the future, we hope to add a two-player competition mode, optimize the interface, and develop a wider range of advanced training options. The soundtrack currently relies on a stable internet connection. In the future, an offline training mode could be added to fulfill the goal of training anytime and anywhere. Note that adding an experimental group and a control group for medical purposes may affect the results, since training specifications for patients will be more standardized and difficult to meet. In the future, the app could be enhanced and thus improve the efficacy of oral training. The current image recognition system could cause slight errors depending on variations in ambient lighting. We hope to improve the accuracy of the image recognition model in the future.

23: Registration number for pilot trial and name of trial registry

This was not applicable as the intervention in this pilot trial was not a clinical or medical treatment for participants. Therefore, this trial was not registered.

24: Where the pilot trial protocol can be accessed, if available

A pilot trial protocol is not available for this pilot trial.

25: Sources of funding and other support (such as supply of drugs), role of funders

This pilot trial was funded by the Taiwanese government, which also audited the study conduct.

26: Ethical approval or approval by research review committee, confirmed with reference number

Approval was granted by the TMU-Joint Institutional Review Board from Taipei Medical University. The number of the ethics committee is No. N202109022. Our study was planned, carried out, analyzed, and interpreted independently of any industrial partners. All participants provided written informed consent before the trial took place.

Reference

Browne R. H. (1995). On the use of a pilot sample for sample size determination. *Statistics in medicine*, *14*(17), 1933–1940. https://doi.org/10.1002/sim.4780141709

Kieser, M., & Wassmer, G. (1996). On the use of the upper confidence limit for the variance from a pilot sample for sample size determination. Biometrical journal, 38(8), 941-949.
